# A CRISPR base editing approach for the functional assessment of telomere biology disorder-related genes in human health and aging

**DOI:** 10.1007/s10522-024-10094-x

**Published:** 2024-02-04

**Authors:** Gustavo Borges, Yahya Benslimane, Lea Harrington

**Affiliations:** 1 Departments of Medicine and Biochemistry and Molecular Medicine, Molecular Biology Programme, Institute for Research in Immunology and Cancer, University of Montreal, Montreal, QC H3T 1J4 Canada; 2 Department of Biochemistry, Temerty Faculty of Medicine, University of Toronto, Toronto, ON M5G 1L7 Canada

**Keywords:** Telomere biology disorders, Telomerase reverse transcriptase, CRISPR Base editing, Telomerase inhibitor resistance

## Abstract

Telomere Biology Disorders (TBDs) are a group of rare diseases characterized by the presence of short and/or dysfunctional telomeres. They comprise a group of bone marrow failure syndromes, idiopathic pulmonary fibrosis, and liver disease, among other diseases. Genetic alterations (variants) in the genes responsible for telomere homeostasis have been linked to TBDs. Despite the number of variants already identified as pathogenic, an even more significant number must be better understood. The study of TBDs is challenging since identifying these variants is difficult due to their rareness, it is hard to predict their impact on the disease onset, and there are not enough samples to study. Most of our knowledge about pathogenic variants comes from assessing telomerase activity from patients and their relatives affected by a TBD. However, we still lack a cell-based model to identify new variants and to study the long-term impact of such variants on the genes involved in TBDs. Herein, we present a cell-based model using CRISPR base editing to mutagenize the endogenous alleles of 21 genes involved in telomere biology. We identified key residues in the genes encoding 17 different proteins impacting cell growth. We provide functional evidence for variants of uncertain significance in patients with TBDs. We also identified variants resistant to telomerase inhibition that, similar to cells expressing wild-type telomerase, exhibited increased tumorigenic potential using an in vitro tumour growth assay. We believe that such cell-based approaches will significantly advance our understanding of the biology of TBDs and may contribute to the development of new therapies for this group of diseases.

**Supplementary Information:**

The online version contains supplementary material available at 10.1007/s10522-024-10094-x.

## Introduction

Telomere biology disorders (TBDs) comprise a group of rare diseases whose distinguishing feature is the presence of short and/or dysfunctional telomeres. As first predicted by Alexey Olovnikov, telomere erosion occurs in cells without a means to replenish telomeres, called the “Theory of Marginotomy” (Olovnikov 1971, 1973). Olovnikov not only recognized the inability of template-dependent DNA polymerases to replicate completely to the 5’ end of a DNA strand (a concept separately recognized by James Watson during T7 DNA concatamer replication (Watson 1972), he also presciently foresaw how multiple rounds of incomplete DNA replication could lead to telomere exhaustion and cellular aging (Olovnikov 1971, 1973). This prediction that telomeres would gradually erode in cell culture was borne out in 1990, when telomeres in primary fibroblasts were observed to shorten during propagation in vitro (Harley et al. 1990), thus providing a molecular mechanism for the limited replicative capacity first described in the 1960’s by Leonard Hayflick (Hayflick and Moorhead 1961; Levy et al. 1992). Eroded telomeres were also shown to be a hallmark of cancer cells (de Lange et al. 1990; Hastie et al. 1990). It is now appreciated that germline mutations in genes responsible for proper telomere length regulation/homeostasis can trigger an accelerated telomere shortening or instability, leading to premature aging phenotypes (Garcia et al. 2007; Shay and Wright 2004; Vulliamy et al. 2001a). The most common TBDs are related to bone marrow failure syndromes (dyskeratosis congenita, Hoyeraal-Hreidarsson syndrome, myelodysplastic syndrome), idiopathic pulmonary fibrosis, liver diseases (liver cirrhosis, non-alcoholic fatty liver diseases, and alcoholic liver diseases), among others (reviewed in Calado and Young 2009; Garcia et al. 2007; Revy et al. 2022).

Since the first identification of the *DKC1* gene as a gene related to a TBD (Heiss et al. 1998) (dyskeratosis congenita), Dokal’s group also detected that dyskeratosis congenita patients exhibited very short telomeres (Vulliamy et al. 2001b). This finding led to the pursuit of additional genes that could impact telomere homeostasis in dyskeratosis and other bone marrow failure syndromes. The most extensively studied genes related to TBDs are from the catalytic core of telomerase, the enzyme responsible for the elongation of telomeres. The main catalytic core of human telomerase is composed of the telomerase reverse transcriptase (hTERT) and the telomerase RNA component (hTR, encoded by *hTERC*) (Feng et al. 1995; Harrington et al. 1997; Kilian et al. 1997; Meyerson et al. 1997; Nakamura et al. 1997; Nakayama et al. 1998). Alterations in hTR were first identified in patients with autosomal dominant dyskeratosis congenita (Vulliamy et al. 2001a), while mutations in human TERT were identified in a subset of patients with acquired aplastic anemia (Yamaguchi et al. 2005). As a general rule, the missense pathogenic variants identified in hTERT and hTR lead to reduced telomerase activity. Thus, these variants are thought to exacerbate the telomere attrition rate in the tissues of the patients affected by TBDs. To date, identifying pathogenic variants connected to a TBD has employed the sequencing of samples obtained from patients and their family members. As reviewed by Revy et al., there are numerous genes involved in TBDs, with significant variability in clinical presentation (Revy et al. 2022).

TBDs are considered rare diseases. As such, alterations in one of the genes implicated in TBDs are rare. Indeed, most (if not all) of our knowledge for classifying these variants comes from sequencing samples from probands affected by one of the TBDs. According to the American College of Medical Genetics (ACMG) classification, for a variant to be considered “pathogenic,” there must be met some criteria based on: population-based data (e.g. is this variant commonly found in healthy subjects?); in silico data (e.g. according to tools like Polyphen2, CADD, SIFT, and others, is this variant predicted to damage the structure or activity of the given protein?); functional data (e.g. is there any previous publication showing how these variants affect the biological role of that given protein?); segregation data (e.g. when a such variant is inherited, are the carriers affected by the disease?), among other criteria.

Although numerous variants are assigned as “pathogenic” with respect to their TBD association, an even more significant number of variants are classified as “uncertain significance.” For example, according to the Varsome database (Kopanos et al. 2019) (a search engine for human genomic variation), from the 987 missense variants identified in hTERT, 880 are “variants of uncertain significance.” Despite detecting variants in patients with a potential TBD, their impact on the clinical onset or development of the disease remains uncertain.

Physicians can also measure telomerase activity to assess the potential impact of genetic alterations in hTERT and hTR. Telomerase activity is assessed either by measuring cell extracts from patients (when available) or using an in vitro system employing human cell lines or a cell-free model (such as the rabbit reticulocyte lysate system) and assessed for enzymatic activity levels via a widely used assay termed the Telomere Repeat Amplification Protocol (TRAP). However, these methods are insufficient to uncover the biological impact that a given genetic variant might impart in tissues during an individual’s lifespan. Thus, there is a need for a cell-based approach to engineer different variants and to assess their impact on cell fitness and telomere integrity.

The development of CRISPR for genome editing has been a watershed development in the ability to assess the relationship between genotype and phenotype. A remaining challenge to this method is that the efficiency of the homologous recombination process required for introducing mutations via CRISPR varies dramatically at different genetic loci and between cell lines (Lin et al. 2014; Usher et al. 2022). The hTERT locus is one such loci, with a low editing efficiency (Xi et al. 2015); thus, it remains a challenge to efficiently mutagenize the hTERT endogenous loci. Nonetheless, CRISPR tools have been successfully used to generate knock-outs in telomerase- or telomere-associated genes to study how the cells cope with telomere attrition under different conditions (Benslimane et al. 2021; Kim et al. 2017). Despite these examples, the introduction of point mutations within the *TERT* coding region is still largely unexplored, given that CRISPR is inefficient for the *hTERT* locus (Xi et al. 2015).

In this work, we employed a CRISPR base editing screen in NALM-6 cells to mutagenize the endogenous alleles of 21 genes involved in telomere biology, including *TERT*. This approach enabled us to probe how different gene variants within genes that contribute to TBDs affect the relative cell fitness of human cells. In addition, using this approach, we also uncovered variants of the telomerase reverse transcriptase that are resistant to telomerase inhibition, and we demonstrate the effect of these variants on cell immortalization and tumorigenic potential in vitro.

## Methods

### Cell culture

NALM-6 and A-431 cells were grown in RPMI 1640 medium with 10% FBS (v/v). HEK293T cells were grown in DMEM medium with 10% FBS (v/v). HA5 cells were grown in Alpha MEM medium with 10% (v/v) FBS. All cells were kept at 5% (v/v) CO_2_ and 37 °C and subcultured every 2–3 days. Parental and gene-edited cell lines used in this study were tested for mycoplasma contamination by standard multiplex PCR.

### Library design

We used the Guide Picker tool (Hough et al. 2017) to scan the sense and antisense strands to find all possible NGG PAM sites within the coding region of each of the 21 genes implicated in telomere homeostasis. A set of 6197 sgRNAs sequences that target the coding region of the genes involved in telomere biology and an additional 1000 control sgRNAs (500 non-target and 500 against non-essential genes) were synthesized by chip-based oligo-pool synthesis as 60-mers (Synbio technologies). First, the pool was amplified by PCR and cloned by Gibson assembly into the pLX-sgRNA vector. Then, it was amplified in a plasmid format and later converted to a lentiviral packed in HEK293T cells using the psPAX and VSVg plasmids as performed by Bertomeu and colleagues (Bertomeu et al. 2018).

### Base editing screens

The ABEmax base editor was subcloned from the pCMV-ABEmax plasmid into the pCW-Cas9 (dox-inducible, puromycin resistance marker) to generate a doxycycline-inducible vector. The new pCW-iABEmax plasmid was amplified and used for lentiviral packaging in HEK293T cells using the psPAX2 and VSVg plasmids. Lentiviral transduction was performed by adding Protamine Sulphate (10 µg/mL) to the viral particles and 10^6^ NALM-6 cells in a final volume of 2 mL for 48 h. Then, the cells were selected in puromycin (1 µg/mL) for 9 days. After expanding the polyclonal population, we isolated monoclonal cell lines by limiting dilution. The expression of the FLAG-tagged ABEmax protein was assessed by western blot in the presence of doxycycline (dox; 1–4 µg/mL for the polyclonal population or 3 µg/mL for the NALM6-iABE clonal cells).

The NALM6-iABE cells were transduced with pooled lentivirus library at an MOI of 0.3 and 100 cells per sgRNA. After 9 days of selection with blasticidin (3 µg/mL), 720,000 cells were induced with doxycycline for 7 days. Then, the cells were propagated without doxycycline (in DMSO or BIBR1532). The sgRNA sequences were detected by PCR of genomic DNA, reamplified (using Illumina adapters) and sequenced using the NextSeq 500 instrument (Illumina).

After sequencing, the reads were aligned to the library using Bowtie 2 ((Langmead and Salzberg 2012), default parameters). Then, the normalization, log-transformation and differential expression of the gene counts were performed using the Limma-Voom approach (Law et al. 2014). Differentially expressed sgRNAs were identified using an FDR cutoff of 6% and absolute fold-change > 1. The differentially expressed sgRNAs in BIBR1532 versus DMSO samples were identified using an FDR cutoff of 1% and absolute fold-change > 1.

### In vitro reconstitution of telomerase

A plasmid encoding hTR was linearized by digestion with the EcoRI-HF enzyme (NEB). Next, the T7 transcription reactions (20 µL) were made according to the Megascript T7 transcription kit. Briefly, each reaction contained 2 µg of the linearized DNA template, 10X reaction buffer, rNTPs (25 mM each) and the T7 enzyme mix. After 2 h of incubation at 37 °C, the template was inactivated by adding two units of TURBO DNase and incubated for 15 min at 37 °C. Finally, the hTR was purified according to the PureLink RNA mini kit (Ambion).

The rabbit reticulocyte coupled transcription/translation reactions (RRL) were performed according to the manufacturer’s instructions. Each reaction (50 µL) containing the FLAG-tagged hTERT plasmids (1 µg) was synthesized in the presence of purified hTR (1 µg) and incubated at 30 °C for 3 h.

### Immunoprecipitation

In vitro-reconstituted telomerase holoenzyme (200 µL of Rabbit Reticulocyte Lysate mix as indicated above) was incubated with 40 µL of magnetic anti-FLAG (Sigma) in 600 µL of 2.3x HypoBuffer (23 mM HEPES, 7 mM KCl, 2.3 mM MgCl_2_, 20 U/mL RNase inhibitors, 2 mM 2-mercaptoethanol) overnight at 4 °C. The beads were then washed 3 times using 600 µL of 2.3x HypoBuffer. After the last wash, the beads were resuspended in 20 µL of 2.3x HypoBuffer and snap-frozen in dry ice.

### Telomerase repeat amplification protocol (TRAP)

Telomerase activity measurement was performed in 25 µL reactions. Each reaction was prepared using 5x TRAP buffer (100 mM Tris-HCl pH 8.3, 7.5 mM MgCl_2_, 315 mM KCl, 0.025% Tween-20, 5 mM EGTA, 0.5 mg/mL BSA), TRAP primer mix (ACX primer 10 µM, NT primer 10 µM, TSNT 100 pM), TS primer (10 µM), dNTP mix (10 mM each), 1 units of Green Taq DNA polymerase (GenScript), 2 µL of the sample (anti-FLAG immunoprecipitate) and 2 µL of either DMSO (final concentration of 0.25% v/v) or BIBR1532 (Selleck Biochem). Ten µL of TRAP reactions were later loaded onto a 10% (w/v) 19:1 acrylamide:bis, non-denaturing gel and subjected to electrophoresis for 90 min at 400 V. Telomerase products were visualized by gel staining with a 100 mL solution of 1X SYBR Gold (Invitrogen) for 30 min. The image acquisitions were made using an Amersham Typhoon scanner (cytiva life sciences).

### Quantitative telomerase repeat amplification protocol (qTRAP)

Telomerase activity measurement was performed as previously described with minor modifications (Herbert et al. 2006). Two µL of crude RRL extract were diluted in 198 µL of CHAPS buffer (10 mM Tris-HCl ph 7.5, 1 mM MgCl_2_, 1 mM EGTA, 0.1 mM Benzamidine, 5 mM Beta-mercaptoethanol, 0.5% (w/v) CHAPS, 10% (v/v) glycerol). All samples were run in triplicate using the FastStart SYBR Green 2X mastermix (Roche), 1 mM EGTA, 0.8 µM ACX primer, 0.8 µM TS primer, and 2 µL of -reconstituted telomerase holoenzyme (1:100 dilution) in a final volume of 25 µL. The samples were incubated in the StepOnePlus thermocycler (Applied Biosystems) with the following program: 30 min at 30 °C, 10 min at 95 °C, 40 cycles of 15 s at 90° C and 1 min at 60 °C. Analysis of the Relative Telomerase Activity (RTA) was performed as previously described (Herbert et al. 2006).

### Tumour spheroid formation assay

A-431 cells (5,000 cells per well) overexpressing hTERT-WT or the BIBR1532-resistant variants were seeded into each well of a 96-wells Ultra-Low Attachment Spheroid microplate (Corning) for 24 h at 5% (v/v) CO_2_ and 37 °C. The spheroids were imaged using an inverted microscope (Leica DMiRB inverted), at 10× magnification, and images were acquired using the Retiga EKI camera. The area, circularity and compactness were measured using ImageJ (NIH).

### Western blot

Immunoblotting was performed according to the TGX stain-free method (Bio-Rad). Two microliters of RRL (containing around 150 µg of total lysate), or 10 µL anti-FLAG immunoprecipitate, were resolved on a 7.5% (v/v) TGX stain-free polyacrylamide gel and transferred to a 0.22 μm nitrocellulose membrane. The membranes were blocked with 4% (w/v) milk in TBST before the blot was probed using the primary antibody anti-FLAG in a 1:1000 dilution (Sigma), followed by the incubation with the HRP-conjugated secondary antibody (1:10,000 dilution in 4% milk). The blots were developed by incubation with the SuperSignal West Femto ECL substrate solution, and chemiluminescence was measured using a ChemiDoc MP (Bio-Rad).

### Statistical analysis

Unless otherwise indicated, statistical analyses were performed on PRISM (www.graphpad.com). Statistical significance was carried out with a Student t-test (2 groups), or with ANOVA (more than 2 groups) using the Sidak or Tukey correction for multiple comparisons.

## Results

### Design and generation of the NALM6-iABEmax base editor cell line

**Fig. 1:**
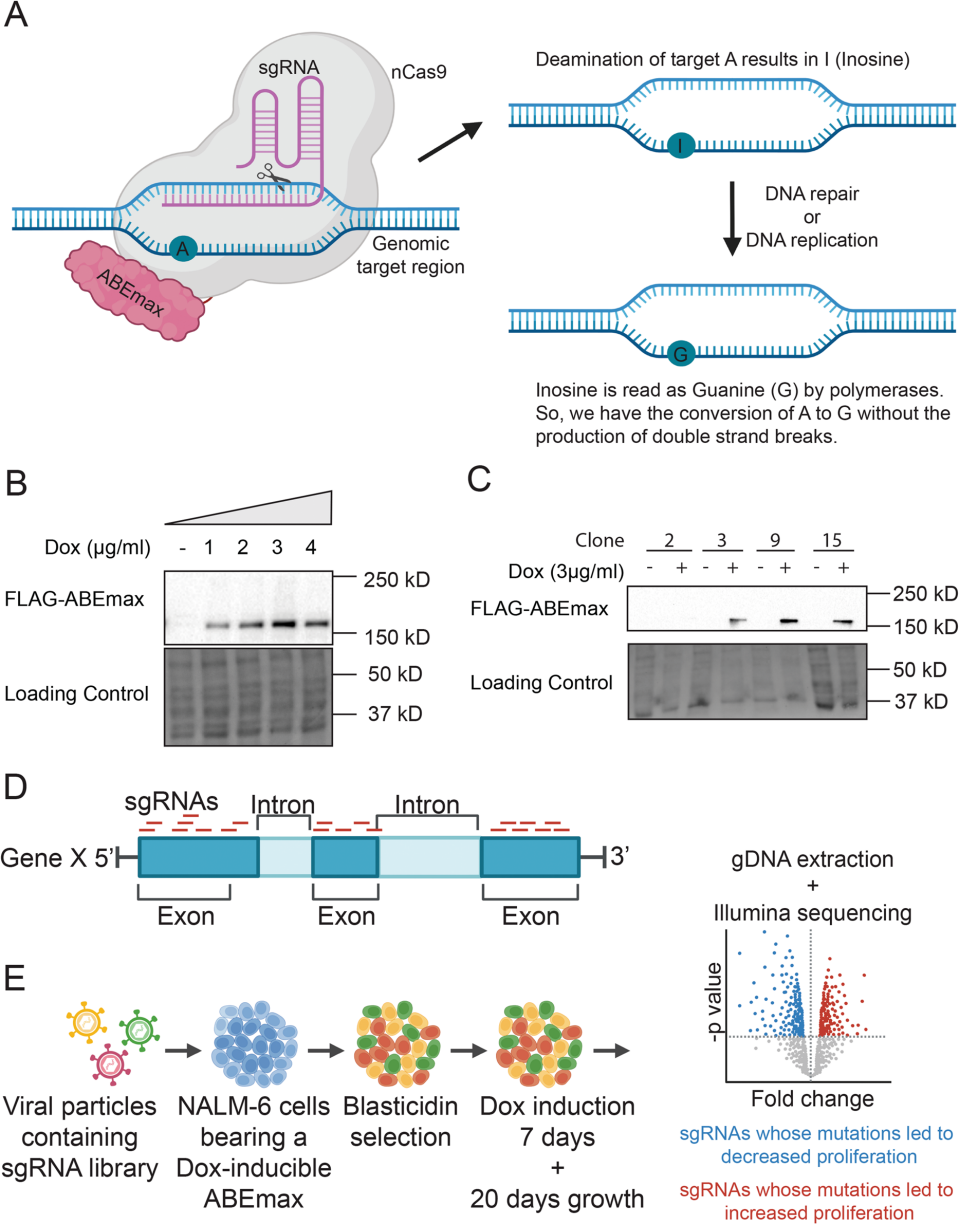
CRISPR Base Editing Screen Library Design.** a **The deamination of the targeted adenosine (A) by the base editor ABEmax results in inosine (I). After DNA repair/replication, inosine is read as guanosine (G) by the polymerases. **b **Polyclonal NALM-6 cells transduced with the dox-inducible base editor ABEmax (NALM6-iABE) were treated with the indicated dose of doxycycline (dox) for 3 days (n = 2). Protein lysates were blotted using FLAG antibody. **c **Clonal NALM6-iABE cells were cultured in the absence or the presence of 3 µg/mL doxycycline for selection of tightly regulated base editor expression (n = 2). **d **Tiling library strategy scheme showing all possible sgRNAs solely against the coding regions of the selected genes. **e** Schematic representation of the CRISPR base editing screen and how to interpret the results

 This study aimed to develop a cell-based system to investigate how genetic perturbations within genes implicated in telomere biology affect cell growth potential. To achieve this objective, we transduced NALM-6 cells, a cell line whose hTERT depletion/inhibition leads to a decreased proliferative capacity (Benslimane et al. 2021) with the doxycycline (Dox)-inducible ABEmax system (Koblan et al. 2018). The NALM-6 cell line is a quasi-diploid pre-B ALL cell line that grows in suspension and has a doubling time of approximately 24 h (Bertomeu et al. 2018). The ABEmax base editing system comprises a Cas9 nickase system fused to a deoxyadenosine deaminase. In brief, this CRISPR-based system can directly convert an adenine (A) into guanine (G) without double-strand break formation (Fig. 1a). The ABEmax editor possesses the ability to convert the adenines located at position 3–11 within the protospacer region (also known as the “activity window”).

To determine the optimal dox concentration for the ABEmax expression, we treated the cells with 1–4 µg/mL dox for three days. First, we performed western blot analysis to detect the FLAG-tagged ABEmax base editor protein (Fig. 1b). Next, we selected a tightly regulated dox-inducible clonal cell line with a high expression of FLAG-ABEmax in the presence of dox for use in our library screens (Fig.1c).

Next, we generated a library containing 6197 sgRNAs, using the Guide Picker tool (Hough et al. 2017) to design all possible sgRNAs targeting the coding region of 21 genes involved in telomere biology (Fig. 1d). In addition, we included 500 sgRNAs targeting non-essential genes in NALM-6 cells and 500 sgRNAs with no sequence match to the human genome (see Supplementary material). Finally, the library was transduced at a low multiplicity of infection (MOI) and selected for lentiviral integration with blasticidin treatment for 9 days.

### Deploying a CRISPR base editing system to identify essential residues in telomere genes

After establishing a cell-based system capable of introducing precise point mutations in different genes related to telomere biology, we decided to investigate further how mutations at those genes could impact cellular growth and division. In previous work from our lab, Benslimane and colleagues showed that telomerase inhibition in NALM-6 cells resulted in loss of cell fitness (Benslimane et al. 2021). Therefore, we predicted that loss-of-function variants will lead to a reduction in cell fitness. Conversely, gain-of-function variants should lead to increased cell fitness.

**Fig. 2:**
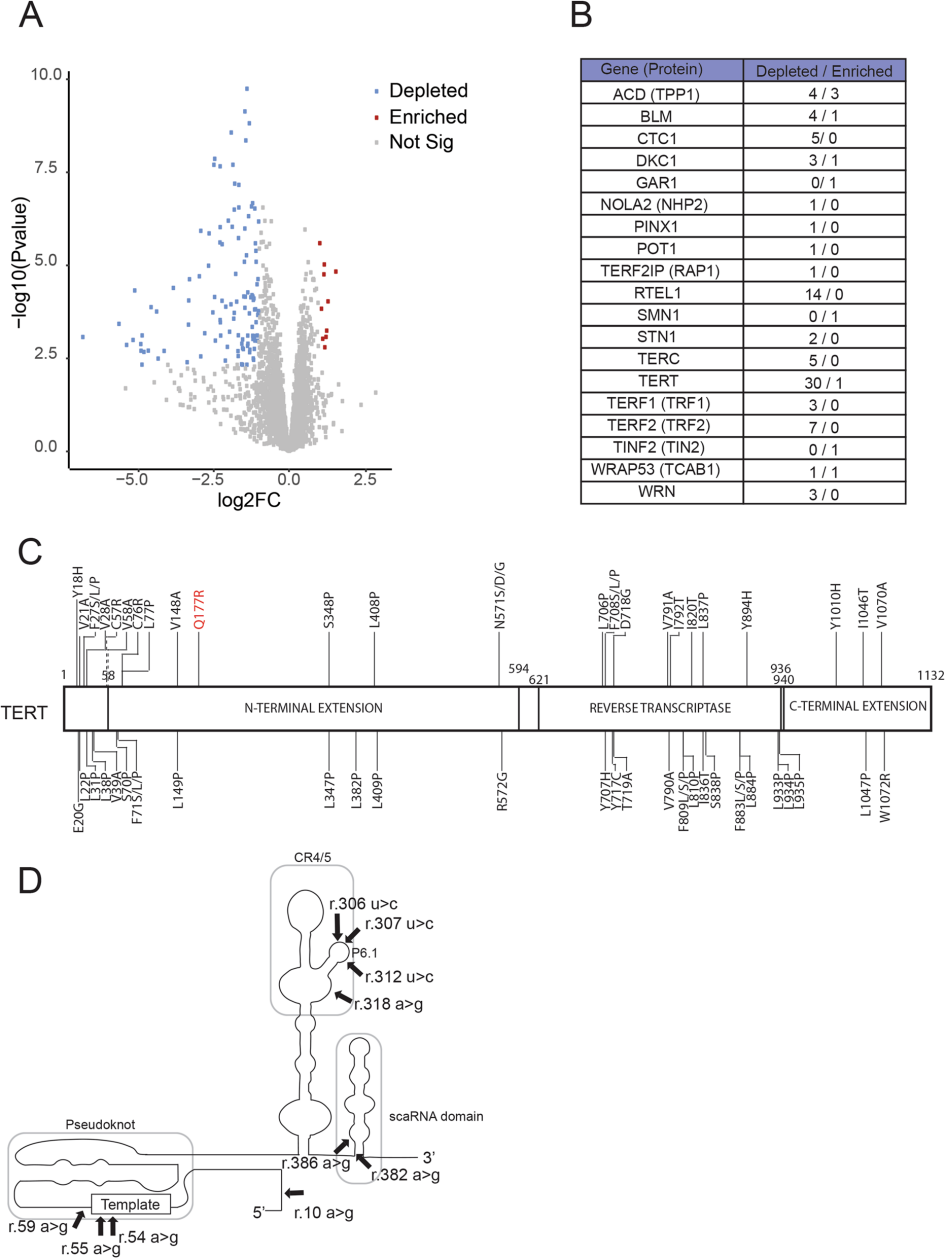
Base Editing Screen to Identify the Essential Residues in *hTERT*and *hTERC* Genes.** a** Volcano plot shows the log_2_ fold change (log_2_FC) and adjusted p-values for all sgRNAs present in our library. The blue dots represent the sgRNAs that were depleted, the red dots represent the sgRNAs that were enriched, and the gray dots represent the sgRNAs that did not exhibit a significantly altered abundance in the late versus early passage cells. The frequency of each sgRNA was considered statistically different when their log_2_FC > 1 and FDR < 0.06. **b** A table containing the total number of sgRNAs identified as enriched or depleted in our screen per gene. **c** A schematic representation of the linear structure of the human TERT protein (NP_937983.2) containing the residues targeted by the sgRNAs against the *hTERT* gene. The variants shown in black are the predicted loss-of-function variants that would be generated by their respective sgRNA. Red indicates the variant predicted to be generated by the *hTERT* sgRNA that was enriched in late passage cells compared to the early passages. The three principal domains of human TERT are depicted. The numbers indicate the respective amino acid residue. **d** A schematic representation of the secondary structure of hTR containing the predicted loss-of-function variants generated by the sgRNAs against the *TERC* gene (NG_016363.1) that were depleted in late passage cells. The three conserved domains (pseudoknot, CR4/5 and scaRNA domain) are shown in rounded rectangles. For further information regarding the hits, please see Supplementary material

To uncover the essential residues of the different telomere biology genes, we cultured the NALM6-iABEmax cells containing the sgRNA library for seven days in the presence of doxycycline to activate the ABEmax expression. Then, the cells were propagated for 20 days in complete media without doxycycline. By sequencing and comparing the abundance of each sgRNA during early (day 0–6) and late passages (day 15–20), we assessed the enrichment or the depletion of sgRNAs over the specified time period (Fig. 1e). Using this approach, we identified 85 sgRNAs that became depleted in the late passages of the NALM6-ABEmax cells and ten sgRNAs that became enriched at late passages (Fig. 2a, b).

Within the catalytic core components of telomerase (hTERT and hTR), the screen identified 30 sgRNAs targeting the *hTERT* gene (Fig. 2c). The hTERT domains with the highest number of hits were the TEN domain (10 sgRNAs), the RT (10 sgRNAs), and the C-terminal extension (4 sgRNAs). For the *hTERC* gene, we found six sgRNAs that were depleted in the late passage cells compared to the early passage cells. They were all located at critical positions encompassing the template region (at the template alignment region (Feng et al. 1995), the CR4/5 domain (including mutations at the P6.1 loop, a region important for the proper interaction with TERT (Mitchell and Collins 2000; Podlevsky and Chen 2016), and the small Cajal body-associated RNA domain (scaRNA) (Theimer et al. 2007) (Fig. 2d).

**Fig. 3:**
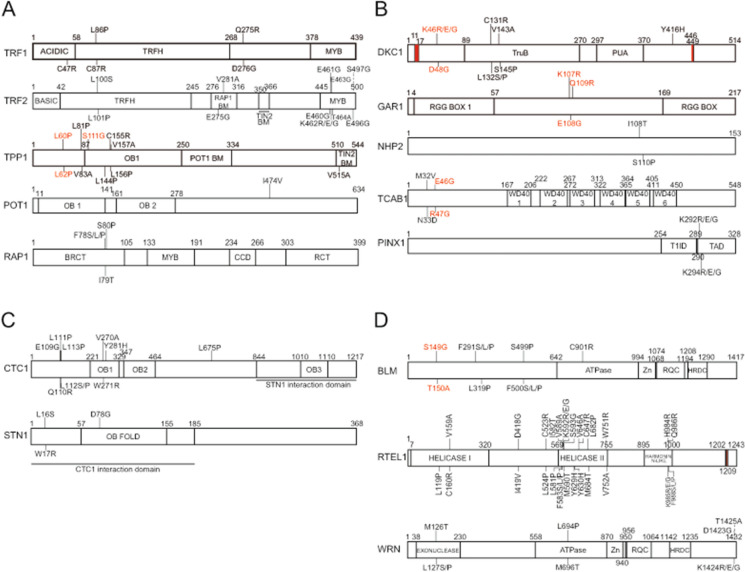
Base Editing Screen to Identify the Essential Residues in Genes Important for Telomere Biology Diseases (TBDs).** a **A schematic representation of the linear structure of the shelterin proteins TRF1 (NP_ 059523.2), TRF2 (AAB81135.1), TPP1 (AAH16904.1), POT1 (NP_056265.2) and RAP1 (NP_061848.2). The variants shown in black are the predicted loss-of-function variants that would be generated by their respective sgRNA. Red indicates the predicted variants that were enriched in late passage cells compared to the early passages. **b **A schematic representation of the linear structure of the telomerase holoenzyme proteins DKC1 (NP_001354.1), GAR1 (NP_061856.1), NHP2 (NP_060308.1), TCAB1 (NP_001137464.1) and the telomerase inhibitor PINX1 (NP_060354.4). The red boxes in DKC1 protein represent the position of both nuclear localization signals. **c **A schematic representation of the linear structure of the proteins CTC1 (NP_079375.3) and STN1 (NP_079204.2), members of the CST complex. **d **A schematic representation of the linear structure of the helicases BLM (NP_001274175.1), RTEL1 (NP_116575.3), and WRN (NP_000544.2)

 For components of the shelterin complex, we identified 16 sgRNAs depleted over 20 days for the shelterin components (Fig. 3). These sgRNAs led to genetic alterations that were located at highly conserved regions of their respective genes (Fig. 3a). For example, within the gene encoding the TRF2 protein, three sgRNAs targeted the myb domain, responsible for the TRF2 binding to the telomeric DNA (Broccoli et al. 1997b). In addition, we found sgRNAs targeting the TRFH domain of TRF1 and TRF2 (Fig. 3a). The TRFH domain is essential for the homodimerization of TRF1 and TRF2 (Bianchi et al. 1997; Broccoli et al. 1997a).

We also examined the other components of the telomerase holoenzyme. We mapped the sgRNAs onto the respective conserved protein domains of their targets (e.g. genetic alterations at the pseudouridine synthase domain (TruB) of DKC1, the proline-rich region of TCAB1, or the transactivation domain of PINX1 (TAD)) (Fig. 3b). Similarly, we identified sgRNAs that targeted the OB-fold domain of the proteins CTC1 and STN1, members of the CST complex (Fig. 3c). Finally, we also uncovered new variants at the BLM, RTEL1 and WRN proteins that might affect cell growth. These results indicated that depleted sgRNAs often targeted conserved domains within their target protein, for example, the helicase domain of RTEL1 or the ATPase domains of BLM and WRN (Fig. 3d).

In addition to the identification of depleted sgRNAs, this cell-based system also identified new variants that enhanced the relative cell fitness of NALM6-ABEmax cells. For example, Fig. 2b shows ten sgRNAs that became enriched in late passage cells (ACD, BLM, DKC1, GAR1, SMN1, TERT, TINF2, WRAP53). These predicted variants are also indicated (in red) in Figs. 2c and 3a–d. Further analysis of individual clonal mutations would be required to confirm the predicted increased fitness conferred to cell lines bearing mutations in the residues shown.

### Identifying alleles that can bypass telomerase inhibition

Despite the inherent inability of based-editing to achieve precise modification of specific amino acids, we nonetheless recognized that this cell-based model might permit the detection of hTERT variants resistant to telomerase inhibition. To test this notion, we chose the small molecule telomerase inhibitor BIBR1532. This small molecule is a potent, selective telomerase inhibitor (Bojovic and Crowe 2011; El Daly and Martens 2007; Pascolo et al. 2002; Ward and Autexier 2005) with a clear, defined mechanism of action (Damm et al. 2001; Pascolo et al. 2002) and with a known binding site in TERT; the Phe-Val-Tyr-Leu (FVYL) pocket (Bryan et al. 2015). Our library contained 50 sgRNAs that targeted this FVYL pocket. We cultured the NALM6-iABE cells containing the sgRNA library as described before; then, the cells were kept in culture for seven days in doxycycline (3 µg/mL) to induce the ABEmax expression, followed by propagation for 20 days in the presence of either DMSO (0.25% v/v) or BIBR1532 at a dosage (30 µM) sufficient to reduce cell proliferation to 50% of the levels obtained in the DMSO-treated controls (Benslimane et al. 2021).

**Fig. 4:**
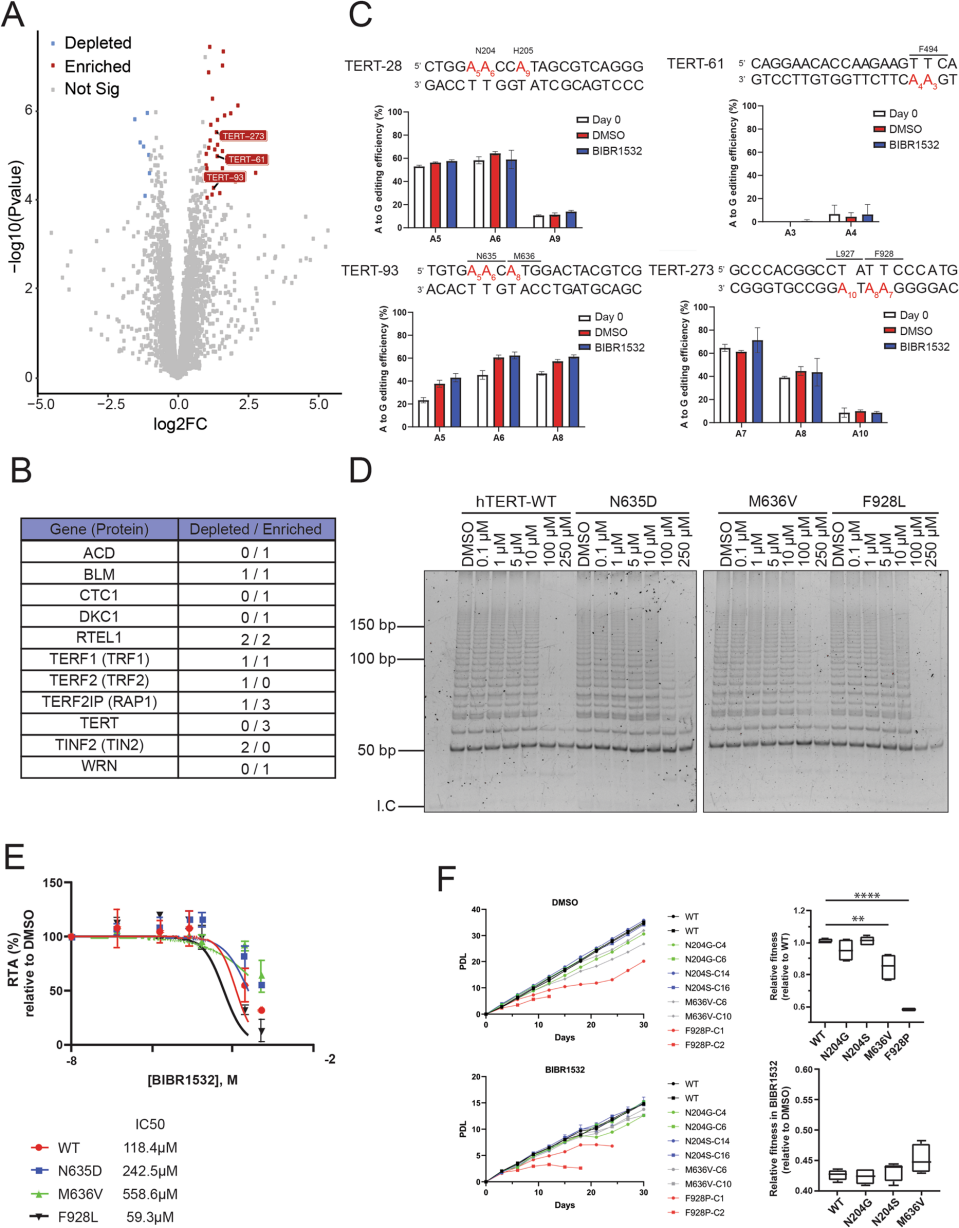
Base Editing Screen Identified BIBR1532-Resistant hTERT variants.** a** A volcano plot showing the changes in sgRNA frequency of NALM6-iABE cells after 20 days of treatment with 30 µM BIBR1532 relative to the respective DMSO control. The *TERT* sgRNAs enriched in the BIBR1532-treated cells are shown in red. **b** A table containing the total number of sgRNAs identified as enriched or depleted in our screen per gene. **c** Percentage of A > G editions for the NALM6-iABE cells individually transduced with the control (TERT-28) or the experimental sgRNAs (TERT-61, TERT-93, and TERT-273) at day 0, or 12 days in DMSO or BIBR1532 (right panel) (*n* = 3). The adenines (in red) are numbered by position in the sgRNA. **d** To confirm the predicted variants are BIBR1532-resistant, we performed in vitro reconstitution of telomerase and assayed the immuno-purified enzyme in the presence of increasing dosages of BIBR1532 (*n* = 3). **e** Quantification of the data shown in **d**, followed by their corresponding IC_50_ values (*n* = 3). The TERT variants N635D and M636V exhibited a higher IC50 than wild-type TERT (potentially resistant), whereas the variant F928L exhibited a lower IC50 (potentially more sensitive). RTA represents the Relative Telomerase Activity (RTA) of samples treated with BIBR1532, compared with samples treated with DMSO. **f** The growth of clonal NALM6-iABE cells (2 clones per genotype) in the presence of DMSO (upper panels) or BIBR1532 (lower panels) was followed for 30 days (left), with relative cell fitness calculated for each variant (comparing growth relative to the same cell clone treated with DMSO, using timepoints at day 27 and 30). PDL represents the population doubling level of the corresponding NALM6-iABE cells

After sequencing, we assessed the enrichment/dropout of each sgRNA by comparing the cells grown in BIBR1532 to those grown in DMSO. We identified 3 sgRNAs targeting the *hTERT* gene (the sgRNAs TERT-61, TERT-93 and TERT-273) that became enriched after BIBR1532 treatment compared to DMSO (Fig. 4a).

To validate these sgRNAs represent *bona fide* hits, we repeated the experiment by individually transducing each of these 3 sgRNAs against *hTERT *into the NALM6-iABE cell line, induced the ABEmax expression using doxycycline, and then cultured the cells with DMSO or BIBR1532 for 12 days. We used a smaller interval for the validation step because introducing the sgRNAs individually into NALM6-iABE cells would allow us to detect a cell fitness advantage faster than in the pooled version (due to decreased noise). A fourth sgRNA, TERT-28, was used as a control as it did not elicit a change in sgRNA frequency after BIBR1532 treatment. After Sanger sequencing of the genomic region covered by these sgRNAs, only two sgRNAs (TERT-93 and TERT-273) exhibited enrichment in the BIBR1532-treated cells compared to their parental control at day 0 (Fig. 4c).

By mapping the specific nucleotides that might have been altered, we then predicted the exact amino acid substitutions. Looking closely at sgRNA TERT-93, only base-editing modifications at nucleotides 5 and 8 showed a slight enrichment compared to the DMSO control (although without statistical significance). The substitution at position 5 would change the codon AAC to GAC, resulting in the substitution of an asparagine (N) for aspartic acid (D) (hTERT-N635D). Similarly, the substitution at position 8 would change the codon ATG to GTG, substituting a methionine (M) for a valine (V) (hTERT-M636V). For the sgRNA TERT-273, the nucleotide at position 7 seemed slightly enriched in the samples treated with BIBR1532 compared to the DMSO control. The mutation at this nucleotide results in substituting a phenylalanine (F) for a serine (S) (hTERT-F928S). To confirm that these point mutations resulted in resistance to BIBR1532 inhibition, we individually introduced them in a plasmid bearing a FLAG-tagged version of human TERT. Then, the plasmids encoding each variant were individually introduced into the rabbit reticulocyte lysate system (RRL) for coupled transcription/translation in vitro. Next, we performed an immunopurification (IP) of the FLAG-tagged hTERT variants using anti-FLAG magnetic beads. Telomerase activity was assessed using the TRAP method (Telomere Repeat Amplification Protocol) in the presence of DMSO or at increasing dosages of BIBR1532 (0.1 to 250 µM). We confirmed that the hTERT variants N635D and M636V exhibited resistance to BIBR1532 in vitro compared to the wild-type (WT) enzyme (Fig. 4d, e). The hTERT variant F928S did not show a detectable level of telomerase activity (Supplementary material, Fig. 2).

Although we cannot rule out the possibility that the F928S variant could be BIBR1532-resistant, the undetectable activity levels would not promote any advantage to cells bearing this mutation. Therefore, we decided to test an additional variant (hTERT-F928L) that could also be generated by modifying the nucleotide 8 from the sgRNA TERT-273. Despite no difference in response to BIBR1532 treatment (Fig. 4b), we included this variant in our analysis. This decision was based on the prior identification of this variant in a cohort of patients with Acute myeloid leukemia (AML) and our goal of assessing the existence of drug-resistant mutations within the human population (Tomlinson et al. 2021). As seen in Fig. 4d, e, the variant F928L appeared slightly more sensitive to BIBR1532 inhibition.

The CRISPR base editing approach offered us the unique opportunity to quickly introduce precise mutations at the endogenous locus of the *TERT *gene and study the effects of these mutations under the control of its native promoter. Following the experiments in Fig. 4b, we decided to isolate clonal cell lines derived from the non-treated cells transduced with the sgRNAs TERT-28 (control), TERT-93 and TERT-273. After clonal isolation and Sanger sequencing of at least 15 clones for each cell line, we picked two homozygous clones from the variants of our interest. We could not identify any positive clones for the N635D, F928S, or F928L variants. Therefore, we tested the ability of the confirmed clones to grow in the presence of either DMSO or BIBR1532. The variants N204S and N204G (derived from the control sgRNA TERT-28) exhibited a growth rate similar to cells carrying the wild-type allele. However, the hTERT variants M636V and F928P showed growth defects in cells treated with DMSO (Fig. 4f, top left and right panel). Notably, only one clone from the F928P survived during the 30-day period we analyzed in the absence of the telomerase inhibitor. Upon treatment with BIBR1532 for 30 days, both clones from the variant F928P failed to grow past 30 days (Fig. 4f, bottom left panel), due to low telomerase activity (Supplementary material, Fig. 3). Cells expressing the hTERT variants N204G, N204S, and M636V retained the ability to proliferate when treated with BIBR1532 (Fig. 4f, bottom left panel), with no significant further impairment in fitness relative to WT hTERT (Fig. 4f, bottom right panel).

### Identification of other potential BIBR1532-resistant variants

**Fig. 5:**
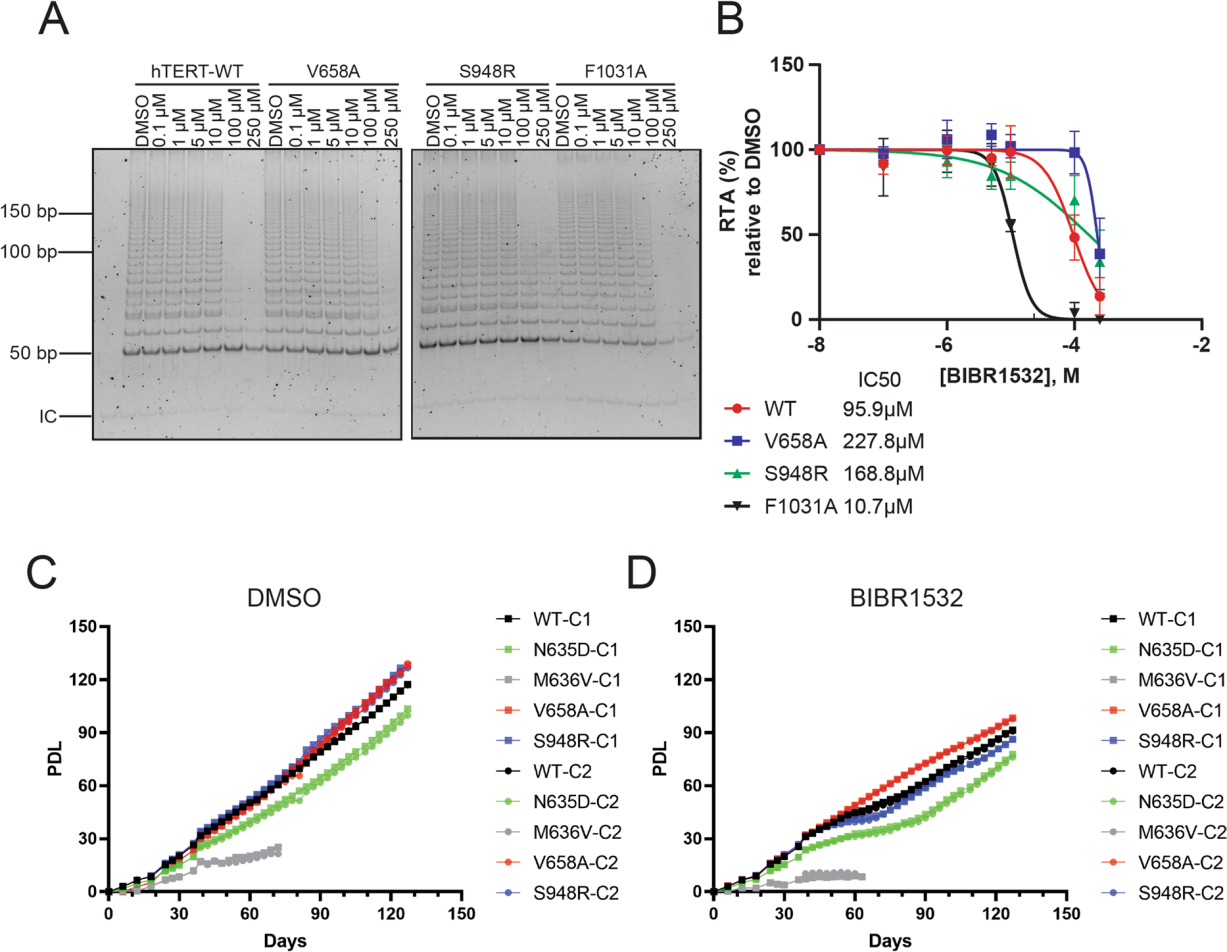
Immortalization potential of BIBR1532-Resistant Variants.** a** Indicated single nucleotide variants were produced in RRL, immunopurified, and tested for BIBR1532 inhibition using the TRAP assay (*n* = 3). IC = PCR internal control with quantification in (**b). c** HA5 cells overexpressing hTERT-WT or the variants N635D, V658A and S948R upon long term culture (2 clones per genotype), treated with 0.25% v/v DMSO. **d** The long-term culture of HA5 cells treated with BIBR1532 (30 µM), including WT hTERT and the hTERT variants indicated (2 clones per genotype)

The telomerase inhibitor BIBR1532 binds to a pocket within hTERT containing the hydrophobic residues FVYL (named FVYL pocket) (Bryan et al. 2015). Therefore, we also wished to assess whether point mutations at hTERT residues within this FVYL pocket that are predicted to interact with BIBR1532 might also influence resistance to BIBR1532. Most variants at the FVYL pocket exhibited a lower telomerase activity, and none exhibited a greater resistance to BIBR1532 (Supplementary material, Fig. 1). Hence, based on our previous findings that the variants N635D and M636V exhibited elevated resistance to BIBR1532, we mapped these residues on the hTERT protein to determine which residues were located nearby. These residues were found to map close to the hTERT catalytic site, with the residue N635 making putative contact with the DNA/RNA heteroduplex (Ghanim et al. 2021). We also searched for alterations near the catalytic site where there was published literature to support a role in activity or processivity. We selected two residues to test based on the cryo-EM structure of human telomerase (Ghanim et al. 2021; Nguyen et al. 2018). These residues are the hyperactive hTERT variant V658A (Xie et al. 2010), located at the motif 3 domain (within the RT domain), and the natural variant S948R (Zaug et al. 2013), located at the beginning of the C-terminal domain near the F928 residue. The S948R variant (rs34062885) is a variant of uncertain significance (VUS). Both variants had prior reports regarding their telomerase activity and processivity (Xie et al. 2010; Zaug et al. 2013), but there was no previous information regarding sensitivity to BIBR1532 or any other telomerase inhibitor. Therefore, we expressed these variants in RRL and performed the IP and TRAP as previously described. Both hTERT-V658A and hTERT-S948R, located close to the catalytic site, exhibited a greater resistance to telomerase inhibition in vitro (Fig. 5a, b).

After identifying the two additional BIBR1532-resistant variants, we decided to test whether these variants would be able to promote cell immortalization. To test this hypothesis, we transduced HA5 cells with hTERT-WT, hTERT-N635D, hTERT-M636V, hTERT-V658A, or hTERT-S948R. The HA5 cells are human embryonic kidney cells transformed with the SV40 small and large T-antigen (Stewart and Bacchetti 1991). As these cells lack hTERT expression, they undergo telomere erosion and eventually enter crisis unless immortalized via ectopic introduction of hTERT (Counter et al. 1998; Stewart and Bacchetti 1991). We thus ectopically expressed hTERT and selected variants to query their immortalization potential (Counter et al. 1998; Stewart and Bacchetti 1991). After hygromycin selection, we isolated clones of cells overexpressing wild-type hTERT or the hTERT variants. After clonal selection (at which point the passage number is set as zero), we compared the number of population doublings with time. We observed that all the variants tested had extended the lifespan of HA5 cells (Fig. 5c). However, the variants N635D and M636V exhibited a growth defect compared to cells expressing the WT, V658A or S948R variants. We also cultured these cells in the presence of BIBR1532 over the same period, and we observed that the HA5 cells expressing the hTERT-V658A variant possessed a slight growth advantage compared to the WT enzyme (Fig. 5d).

The importance of telomerase for tumorigenesis is well known, and the effects of telomerase inhibition in cancer cells are well established (Hahn et al. 1999; Herbert et al. 1999; Zhang et al. 1999). We therefore wished to assess the tumorigenic potential of the BIBR1532-resistant variants. For this purpose, we decided to use the A431 cells, an epidermoid carcinoma cell line, because of (i) its ability to form tumours in nude mice (Adhikary et al. 2013), (ii) its ability to form tumour spheroids in vitro after 24 h (Adhikary et al. 2013), and (iii) the preferred use of 3D cell culture models as an alternative to animal testing. We overexpressed the hTERT-WT and BIBR1532-resistant variants in A431 cells by lentiviral transduction (as previously described for HA5 cells). After hygromycin selection, we seeded the cells into a 96-wells ultra-low attachment plate and waited 24 h for spheroid formation. The overexpression of either the wild-type or hTERT variants was sufficient to increase the compactness of the tumour spheroids compared to the parental A431 cells (Supplementary material, Fig. 4). Although the overexpression of the hTERT variant N635D exhibited an increased spheroid area compared to the parental A431 cells, this increase did not significantly differ from the spheroid area of cells expressing wild-type hTERT (Supplementary material, Fig. 4). These data provide evidence that BIBR1532-resistant variants might have tumorigenic potential that is at least equivalent to wild-type telomerase.

## Discussion

The correlation between gene mutations and the phenotype of someone living with a TBD is complex due to a multitude of factors, such as a lack of clinical data from patients and their family members (due to the low number of samples), as well as a lack of detailed understanding of how these variants affect telomere integrity in a physiologically relevant context. The results from the base editing screen provided a relatively rapid means (20 days) to map the essential regions in numerous genes implicated in telomere biology. This approach has proven useful, as several of the variants we identified that led to reduced cell fitness were previously described loss-of-function alleles within the template region of hTR, the MYB domain of TRF2, or the OB-fold region of TPP1. However, the most notable contribution of this cell-based model is the possibility of studying the impact of variants of uncertain significance (VUS) without overexpression or ectopic expression in different cell lines. For example, we identified the hTR variants r. 54 a > g and r.382 a > g as essential in our initial screen. These variants (also known as rs1288561509 and rs1777958465, respectively) were identified as germline variants in a clinical test for dyskeratosis congenita. Using our cell model, we found that cells bearing these variants had a lower cell fitness and growth rate, suggesting a possible physiological consequence for these VUS.

We note that our base-editing library queried three genes previously studied in genetic studies of human longevity: *hTERT*, *hTERC*, and *hWRN* (see Human Aging Genomic Resources database https://genomics.senescence.info). For example, previous studies on centenarians of Ashkenazi Jewish or Italian descent found two intronic variants and two synonymous variants (A305A, and H1013H) in the *TERT* gene that are enriched in their centenarian populations and postulated to affect *TERT* mRNA expression (Atzmon et al. 2010). The variants associated with longevity in the *hTERC* gene are also intronic (IVS-99 C > G, and IVS + 12 A > G), and might also affect gene expression (Atzmon et al. 2010). The variants associated with longevity found in the Werner helicase gene are rs13251813 (an intronic variant found in the Danish population), and the L787L synonymous variant (found in American families) (Sebastiani et al. 2012; Soerensen et al. 2012). It is intriguing that rare variants in those genes can cause premature aging syndromes, such as the TBDs (in the case of rare missense/nonsense variants), while some intronic and synonymous variants are enriched in centenarians. In our work, the design of our library allowed us to assess only the coding regions within the genes of interest. In Figs. 2 and 3, for example, we presented an analysis of the sgRNAs that resulted in missense mutations, however the design of the sgRNA library was unbiased and also included sgRNAs that could result in other alterations (e.g. nonsense, silent) (Supplementary material). It is possible that the inclusion of sgRNAs covering the non-coding regions of the same genes of our library would reveal additional variants that could influence gene expression, with potential impacts for human aging.

This NALM6-iABE cell line containing the entire library also permitted the discovery of new properties not previously ascribed to TERT. We identified point mutations in hTERT that were resistant to BIBR1532 inhibition and permitted cellular immortalization and in vitro tumour spheroid formation. We note that all four potentially BIBR1532-resistant variants identified so far are located near the catalytic site of telomerase and not at the predicted binding site of BIBR1532 (the FVYL pocket). Although we tested a significant number of mutations at the FVYL pocket, we cannot rule out the possibility of the involvement of this region since we have not tested all possible modifications in the FVYL pocket. It would also be important to determine if these mutations have consequences for tumour progression in animal models. In addition, it would be interesting to query the genes/networks that permit cells bearing these variants to survive, particularly in the presence of telomerase inhibitors. For example, the discovery of new synthetic-sick-lethal interactions might prevent the emergence of drug resistance. Further studies will be needed to unveil what mechanisms might be exploited to sensitize cells bearing hTERT variants or other telomere-associated protein variants.

This cell-based screen also revealed potential genetic alterations in eight other genes that appeared to confer resistance to BIBR1532 (Supplementary material). It is important to note that the cell fitness of cells treated with 30 µM BIBR1532 will reflect both on-target and off-target effects of BIBR1532 at this concentration. Without further verification of these variants using the same methods employed in this study for hTERT, we cannot confirm whether these are real ‘hits’, i.e. whether resistance to BIBR1532 is conferred by genes unlinked to hTERT. Additionally, as we did not confirm the frequency of biallelic editing at each targeting site, our results will also be influenced by variations in editing frequency at different loci. Notwithstanding these potential limitations, it remains possible that alterations of the interaction of telomerase with its substrate, i.e. the telomere, could be affected by telomere- or telomerase-associated gene products in a manner that would buffer against the inhibition of telomerase activity in cells. Additional studies will be necessary to decipher if the hits identified are truly BIBR1532-resistant and what is the molecular mechanism behind this potential resistance.

To our knowledge, this data provides the first direct evidence of an *hTERT* allele conferring an inherent resistance to a small molecule. In addition to the ability to model variants related to TBDs or drug-resistance, the methods employed here are modular. They can be applied not only to any putative telomerase inhibitor but also to other compounds (e.g. G-quadruplex stabilizing ligands) or even be used in different conditions, e.g. upon exposure to agents that induce replication or oxidative stress or in cells expressing non-functional p53.

The validation of the hits from the CRISPR base editing screen used to identify variants resistant to BIBR1532 showed that only one sgRNA, TERT-93, was truly a hit. The low number of hits might raise the question as to whether such low numbers are seen because of a low on-target efficiency of the base editor used or because of a low on-target sgRNA activity. A brief review of the data in Fig. 4C appears to argue against ABEmax as the source of the efficiency problem as we have a different percentage of A > G conversion depending on the sgRNA. Thus, it is very likely that factors such as sgRNA sequence, structure, and even the positioning of the target adenine within the protospacer region of the sgRNA might have a bigger impact.

In conclusion, our study presents a valuable tool for the identification of new pathogenic variants, and for studying the long-term impact of such variants on the genes involved in TBDs. Using our CRISPR base editing model we identified key residues that impact cell fitness and growth, we provided functional evidence for VUS in patients with TBDs, and we identified variants resistant to telomerase inhibition. Also, the use of the base editor proved to be a suitable tool for making single nucleotide changes to endogenous *hTERT*, a difficult-to-modify gene. Our data support base editing as an emerging and potential strategy for cell-based modulation of TBD and other diseases.

### Supplementary Information

Below is the link to the electronic supplementary material.

Supplementary material 1 (DOCX 1326.1 kb)

Supplementary material 2 (XLSX 24.4 kb)

Supplementary material 3 (XLSX 223.0 kb)

## Data Availability

Data is provided within the manuscript or supplementary information files.
