# Supplementary material for: A CRISPR base editing approach for the functional assessment of telomere biology disorder-related genes in human health and aging
Source: Biogerontology. 2024 Feb 4;25(2):361–78. doi: 10.1007/s10522-024-10094-x (PMC10998809; doi:10.1007/s10522-024-10094-x)
Supplement: Supplementary file 1 — Supplementary material 1 (DOCX 1326.1 kb) [file 10522_2024_10094_MOESM1_ESM.docx]

## Supplementary Information

**Supplementary Figure 1. Impact of hTERT Mutations on Recombinant Telomerase Produced in Rabbit Reticulocyte Lysates (RRL) and Their Sensitivity to the Telomerase Inhibitor BIBR1532. a.** Schematic representation of the *hTERT* gene (NG_009265.1) with the three main domains (N-terminal extension, reverse transcriptase (RT), and thumb domains) highlighted. **b.** Quantitative PCR-based TRAP assay (qTRAP) analysis of the telomerase activity from the FVYL pocket (the predicted BIBR1532 binding site) variants tested. **c.** Quantitative TRAP analysis of the telomerase activity after treatment with control (0.3125% v/v DMSO) or 312.5 µM BIBR1532 of selected FVYL pocket variants. Data represented is already normalized by their respective DMSO control. Data are represented as mean $\pm$ SD. Data represent n=3 biological replicates. Statistical analysis was performed using one-way ANOVA.

**Supplementary Figure 2. Telomerase activity of hTERT variants M636V and F928S**. Indicated single nucleotide variants were produced in RRL, immunopurified, and tested for BIBR1532 inhibition using the TRAP assay (n=3). IC = PCR internal control.

**Supplementary Figure 3. Telomerase Activity of NALM6-iABE Clonal Cells.** Telomerase activity of 2 clones of each genotype was assessed using the quantitative TRAP (qTRAP) method. The lower telomerase activity of the variants hTERT-M636V and hTERT-F928P correlates with the lower proliferation rate exhibited by both variants. RTA stands to “Relative Telomerase Activity”.

**Supplementary Figure 4. The overexpression of telomerase affects the features of tumour spheroids *in vitro***. A431 cells overexpressing wildtype or indicated variants were plated in ultra-low attachment plates to stimulate the formation of tumour spheroids in vitro. Spheroid area (a), circularity (b), and compactness (c) in parental cells or the A431 cells overexpressing the wildtype telomerase or the indicated hTERT variants (at least 5 spheroids in 3 independent replicates).

**Supplementary Table 1. List of sgRNAs.** Table containing gene list, targeted genomic region, log2 fold change, p-value, False Discovery Rate (FDR) values, and predicted variants from all the sgRNAs identified as hits in the pooled experiments comparing late passage vs early passage NALM6-iABE cells. This table contains sgRNAs that reached our threshold levels to be considered hits (log2FC > 1, FDR < 0.06). The sgRNAs targeting splicing sites or control sgRNAs (against non-targeting and non-essential genes) were excluded from this list and they are also absent from Figure 2b list.

| ***Gene-sgRNA ID*** | ***Targeted genomic region*** | ***Log2FC*** | ***P.Value*** | ***FDR*** | ***Amino Acid Residues Affected*** | ***Predicted variants*** |
| --- | --- | --- | --- | --- | --- | --- |
| STN1-55 | CTTGCAGTGGATGACAGCAC | -6.8101538 | 0.00087426 | 0.0200918 | D78 | D78G |
| WRAP53-25 | TTCCCCGATGAATAAAAATG | -5.6177964 | 0.00038673 | 0.01137402 | M32, N33 | M32V, N33D |
| TRF1-78 | CTCGGCAAAGAGAGAGGCAG | -5.3652405 | 0.00142872 | 0.02684141 | L86, C87 | L86P, C87R |
| WRN-27 | ATGGACAAAACGAAAAGGGG | -5.1518563 | 0.00104986 | 0.02265222 | D1423, K1424, T1425 | D1423G, K1424R/E/G, T1425A |
| TERT-252 | CGTCTTGAGGAGCACCCCGT | -5.0972509 | 4.90E-05 | 0.00320734 | L409 | L409P |
| TERT-642 | CCGTCGTCATCGAGCAGGTC | -4.8956115 | 0.00135312 | 0.02611629 | I792 | I792T |
| TERC-15 | ACCCTAACTGAGAAGGGCGT | -4.8574988 | 0.00483724 | 0.0599821 | r.54, r.55, r.59 | r.54A>G, r.55A>G, r.59A>G |
| BLM-96 | TCATCATCAAATTCAATACA | -4.8518778 | 0.00079245 | 0.01900396 | F291 | F291S/L/P |
| TRF2-96 | AAGGCGCACCGTGAGGAGGT | -4.7932004 | 0.00219888 | 0.0346009 | V281 | V281A |
| ACD-202 | CTACACCCAGCGGATGCAAC | -4.6487126 | 0.00203369 | 0.0333328 | V83, L81 | V83A, L81P |
| ACD-40 | TCACCAGGCATCGGACACTG | -4.5569117 | 0.00013747 | 0.00601353 | C155, L156, V157 | C155R, L156P, V157A |
| TERC-11 | CAGAGCCCAACTCTTCGCGG | -4.373774 | 0.00018037 | 0.00680319 | r.312, r.307, r.306 | r.312 U>C, r.307 U>C, r.306 U>C |
| TRF2-115 | CTGTGATGATTAAGGATCGC | -4.1260692 | 0.00206926 | 0.03341253 | I529, M528 | I529V, M528V |
| CTC1-246 | TGTGCCACACCAGCTGGGCA | -3.8218311 | 4.18E-05 | 0.00287013 | V270, W271 | V270A, W271R |
| WRN-375 | CCATTGGCAGTGCTGTCTTT | -3.3655734 | 0.0041665 | 0.05390386 | L694, M696 | L694P, M696T |
| RTEL1-138 | TCTCCATGACAGGATAGGAA | -3.321654 | 0.00040639 | 0.01179437 | V589, M590 | V589A, M590T |
| TERT-314 | CGCCGCACGAACGTGGCCAG | -3.3129829 | 9.10E-05 | 0.00463386 | V28, F27 | V28A, F27S/L/P |
| TERT-659 | GGGCAGGCGGGGCAACCTGC | -3.2878091 | 2.44E-05 | 0.0019336 | L382 | L382P |
| TERT-153 | ACAAAGTACAGCTCAGGCGG | -2.6585532 | 1.06E-05 | 0.00108855 | F708, Y707, L706 | F708S/L/P, Y707H, L706P |
| TERT-45 | TCTTGAGGAGCACCCCGTAG | -2.64022 | 1.43E-06 | 0.00024318 | L408, L409 | L408P, L409P |
| TERT-277 | CCCCAGGCGCCGCACGAACG | -2.4816994 | 2.04E-08 | 1.23E-05 | L31 | L31P |
| TERT-127 | CGCGTACGACACCATCCCCC | -2.4577861 | 7.33E-05 | 0.00411641 | T719, D718, Y717 | T719A, D718G, Y717C |
| NHP2-28 | TTAGAGGGGATATAGACATA | -2.4543826 | 1.41E-08 | 1.16E-05 | I108, S110 | I108T, S110P |
| TERC-6 | TGTCAGCCGCGGGTCTCTCG | -2.4339501 | 0.00109229 | 0.02303139 | r.318 | r.318 A>G |
| RTEL1-695 | TTCTCCAGGCAGACTGGGAA | -2.2817327 | 2.50E-06 | 0.00037219 | C523, L524 | C523R, L524P |
| CTC1-296 | CATAGGCTGTACCAGGCCGA | -2.2763872 | 2.24E-08 | 1.23E-05 | Y281 | Y281H |
| TERT-143 | AGCCACTGCACGGCCTCGGA | -2.2762341 | 0.00030558 | 0.00991103 | W1072, V1070 | W1072R, V1070A |
| BLM-38 | TGTCACATTCTCGCCTGGAG | -2.2747474 | 9.69E-07 | 0.00019939 | C901 | C901R |
| TERF2IP-6 | CGTGGAGATGAAATCACCCG | -2.2573007 | 0.00079928 | 0.01900396 | F78, I79, S80 | F78S, I79T, S80P |
| ACD-65 | AAGCAGCGTGGCCCCGACGT | -2.2102979 | 2.79E-06 | 0.00037279 | L144 | L144P |
| TERT-422 | AGAACAGGCTCTTTTTCTAC | -2.1044909 | 0.00377074 | 0.05056617 | R572, N571 | R572G, N571S/D/G |
| TRF2-36 | AACCCGCAGCAATCGGGACA | -2.0066918 | 6.49E-07 | 0.00015341 | L101, L100 | L101P, L100S |
| TERT-416 | AGGGCACGCACACCAGGCAC | -1.9185307 | 0.00011535 | 0.00542797 | V58, C57 | V58A, C57R |
| CTC1-300 | TGCTCAGCTCCTTCCAGGAA | -1.9093203 | 2.76E-09 | 3.41E-06 | L675 | L675P |
| TERT-683 | GCGGAAGGAGGGGGCGGCGG | -1.8770861 | 9.49E-07 | 0.00019939 | F71, S70 | F71S/L/P, S70P |
| TERT-182 | TCCTTCAGGCAGGACACCTG | -1.8106416 | 3.27E-07 | 9.50E-05 | L77, C76 | L77P, C76R |
| TERT-299 | TGGAGAGGATGGAGCCCTGC | -1.7950967 | 6.56E-08 | 3.17E-05 | S838, L837, I836 | S838P, L837P, I836T |
| TERC-97 | TGCGGAGGGTGGGCCTGGGA | -1.7301396 | 9.78E-05 | 0.00478475 | r.10 | r.10 A>G |
| BLM-64 | TTACCTAAGGCATTTTGAAG | -1.6873464 | 0.00209317 | 0.03368851 | L319 | L319P |
| TRF1-2 | ACTTCTCAAGATAAACCTAG | -1.6796212 | 0.00284684 | 0.04173956 | Q275, D276 | Q275R, D276G |
| TERT-48 | AGCCATACTCAGGGACACCT | -1.6702005 | 1.91E-06 | 0.00031434 | Y894 | Y894H |
| RTEL1-53 | AGAAGAGCCTGGAGTTCTGG | -1.658847 | 2.87E-07 | 9.44E-05 | K592, S593 | K592R/E/G, S593G |
| TERT-94 | CCAGCACGTCGTCGCCCACG | -1.6585047 | 7.06E-08 | 3.17E-05 | L149, V148 | L149P, V148A |
| DKC1-41 | TTGTGACTTCACCAAGCGAG | -1.5684238 | 0.0047367 | 0.05920038 | V143, S145 | V143A, S145P |
| DKC1-9 | AACATACTCCTGCTTCCAGG | -1.5593078 | 0.0013238 | 0.02597092 | Y416 | Y416H |
| TRF2-20 | TGAAGAAAAGGAGACTTGGG | -1.5345818 | 0.00174239 | 0.03052884 | K462, E461, E460 | K462R/E/G, E461G, E460G |
| TERT-86 | AGCGTAGGAAGACGTCGAAG | -1.5331143 | 0.00101691 | 0.02223246 | L810, F809 | L810P, F809S/L/P |
| RTEL1-574 | TGTGAGTTGCGAGTGGGTCC | -1.4929813 | 7.10E-05 | 0.00408933 | L119 | L119P |
| TERT-139 | CCAGCAGCAGGCCGCACCAG | -1.4752872 | 8.28E-06 | 0.00092951 | L935, L934, L933 | L935P, L934P, L933P |
| TERT-645 | CTGCACCAGCCGCCAGCCCT | -1.458079 | 7.48E-10 | 1.85E-06 | V39, L38 | V39A, L38P |
| TERT-285 | TGCACCAGCCGCCAGCCCTG | -1.4578687 | 0.00014313 | 0.00616838 | V39, L38 | V39A, L38P |
| TRF2-53 | AGAAGAAAGCGAGTGGGTCA | -1.4437512 | 7.22E-05 | 0.0040994 | S497, E496 | S497G, E496G |
| TERC-90 | ACGGAGCGAGTCCCCGCGCG | -1.4273239 | 4.52E-09 | 4.46E-06 | r.382, r.386 | r.382 A>G, r.386 A>G |
| DKC1-92 | ATTAAACAACCAGTCACCTT | -1.4064066 | 5.55E-06 | 0.00065254 | C131, L132 | C131R, L132S/P |
| STN1-28 | CAAACCCCACAAGAGGGAAG | -1.3916506 | 0.00485241 | 0.0599821 | W17, L16 | W17R, L16S |
| TERT-260 | CCCTGATGCGCACGGCGTGG | -1.3699197 | 0.00151045 | 0.02795185 | I820 | I820T |
| ACD-38 | CACCAGGCATCGGACACTGT | -1.3622275 | 5.52E-05 | 0.00345011 | L156, C155 | L156P, C155R |
| TERT-89 | ACCTGAGGAAGGTTTTCGCG | -1.3581774 | 0.00132456 | 0.02597092 | L884, F883 | L884P, F883L/S/P |
| TERT-134 | AGAGAGCTGAGTAGGAAGGA | -1.3357194 | 4.92E-07 | 0.00013507 | S348, L347 | S348P, L347P |
| RTEL1-164 | CACACACACCTTAGGCCTGG | -1.3095938 | 0.00020277 | 0.00736679 | V159, C160 | V159A, C160R |
| TRF1-105 | CCTGGCACTCGAGCAGTTCC | -1.3004251 | 0.00224581 | 0.03522717 | C47 | C47R |
| BLM-355 | TACAAAGGACTTCTGTAAAT | -1.2934889 | 0.00192042 | 0.03248304 | S499, F500 | S499P, F500S/L/P |
| TERT-548 | CTACCGCGAGGTGCTGCCGC | -1.2622075 | 7.49E-05 | 0.00415603 | E20, Y18 | E20G, Y18H |
| TERT-399 | GGCAGGCGGGGCAACCTGCG | -1.2392358 | 2.69E-07 | 9.44E-05 | L382 | L382P |
| TERT-516 | GCGGCAGCACCTCGCGGTAG | -1.2236024 | 6.56E-05 | 0.0039533 | L22, V21 | L22P, V21A |
| RTEL1-626 | ACGCACCCAGGAGGGCAGTT | -1.2028383 | 2.20E-07 | 9.04E-05 | W751, V752 | W751R, V752A |
| RTEL1-424 | CCTCAAACTGCTGCTTATGG | -1.1943598 | 7.12E-05 | 0.00408933 | F988 | F988S/L/P |
| WRN-199 | AAGCAACATTTTTAATCCCT | -1.1920318 | 0.00098728 | 0.0216806 | M126, L127 | M126T, L127S/P |
| RTEL1-91 | TGGCGGACATTATCCAGGTG | -1.179491 | 8.79E-05 | 0.00453263 | D418, I419 | D418G, I419V |
| TRF2-12 | CGCCGAGCCCTACCTCCTCA | -1.1703251 | 0.00358885 | 0.04884984 | E275 | E275G |
| RTEL1-689 | CATAGTAAGCACTGATGGTC | -1.1605231 | 8.07E-05 | 0.00428771 | Y629, Y630 | Y629H, Y630H |
| POT1-102 | AGTGTAATTCCTGTGAGATC | -1.1582922 | 0.00096834 | 0.02151057 | I474 | I474V |
| PINX1-66 | CTGAAGCCCAAAAAGAGGAG | -1.146925 | 0.00182567 | 0.03154062 | K292, K294 | K292R/E/G, K294R/E/G |
| CTC1-682 | CGAGAGCAGCTGTTACTTTT | -1.1415416 | 0.00082153 | 0.0192378 | Q110, E109 | Q110R, E109G |
| RTEL1-525 | AAGAAGATCAGGAGCCCATA | -1.1365177 | 0.00015903 | 0.00638423 | L581, I582, F583 | L581P, I582T, F583L/S/P |
| RTEL1-342 | CCGGCAGACCGCCAGGAAGG | -1.1116131 | 2.64E-06 | 0.00037219 | V646, C647 | V646A, C647R |
| RTEL1-45 | CCATAAGCAGCAGTTTGAGG | -1.1068376 | 0.00015653 | 0.00635598 | H984, K985, Q986 | H984R, K985R/E/G, Q986R |
| TERT-303 | ACCTGTACGCCTGCAGCAGG | -1.0946002 | 4.20E-06 | 0.00050653 | Y1010 | Y1010H |
| RTEL1-36 | TGCATCTTGAGGACAACCCG | -1.0882873 | 0.00034996 | 0.01077109 | L682, M684 | L682P, M684T |
| TERT-216 | CTTTCAGGATGGAGTAGCAG | -1.0870587 | 8.28E-06 | 0.00092951 | L1047, I1046 | L1047P, I1046T |
| CTC1-94 | AAAAGTAACAGCTGCTCTCG | -1.0437277 | 0.00110497 | 0.02306511 | L113, L112, L111 | L113P, L112S/P, L111P |
| TRF2-184 | AAAGGAGACTTGGGTGGAAG | -1.0320058 | 0.00022323 | 0.00782239 | T464, E463, K462 | T464A, E463G, K462R |
| RTEL1-595 | TTCTCCATGACAGGATAGGA | -1.0252493 | 2.42E-05 | 0.0019336 | V589, M590 | V589A, M590T |
| TERT-78 | ATGACGACGGCATCCCTCAG | -1.0065662 | 0.00018599 | 0.00696209 | V791, V790 | V791A, V790A |
| TERT-284 | CAGCCACTGCACGGCCTCGG | -1.0038601 | 6.83E-07 | 0.00015341 | W1072, V1070 | W1072R, V1070A |
| ACD-44 | CGAAGGAGCTTCCTCTTCCG | 1.01579315 | 2.62E-06 | 0.00037219 | L60, L62 | L60P, L62P |
| ACD-135 | ACACCCTCCAGTCCACGAGC | 1.07006459 | 0.00015172 | 0.00629967 | S111 | S111G |
| ACD-2 | CAGCTTGGACCCGAGCACAG | 1.11210401 | 0.00097518 | 0.02151057 | V515 | V515A |
| GAR1-58 | CAAAGAACAAATTGGAAAAG | 1.15171058 | 1.80E-05 | 0.00165091 | K107, E108, Q109, I110 | K107R, E108G, Q109E, I110V |
| TERT-571 | CCAGCTCGGCGCTGCCACTC | 1.16400856 | 9.77E-06 | 0.00104996 | Q177 | Q177R |
| DKC1-11 | CTAAGTTGGACACGTCTCAG | 1.18141768 | 0.00165139 | 0.02956342 | K46, D48 | K46R/E/G, D48G |
| TINF2-3 | AGCAATCCAAGCAATTCCTG | 1.22118138 | 0.0008689 | 0.02006185 | C359, L357 | C359R, L357S/P |
| SMN1-77 | TTATTAGCTACTTCACAGAT | 1.24377612 | 0.00059001 | 0.01567336 | V148 | V148A |
| WRAP53-227 | CCGAAAGGGGGGATCCGCCC | 1.28698423 | 9.62E-05 | 0.00475277 | E46, R47 | E46G, R47G |
| BLM-273 | CTTTAAGTACCATCAATGAT | 1.5452055 | 1.52E-05 | 0.00149979 | S149, T150 | S149G, T150A |

**Supplementary Table 2.** List of sgRNAs differentially expressed in NALM-6-iABE cells treated with BIBR1532 or DMSO. Table containing gene list, targeted genomic region, log2 fold change, p-value, False Discovery Rate (FDR) values, and predicted variants from all the sgRNAs identified as hits in the pooled experiments comparing NALM6-iABE cells treated with BIBR1532 vs DMSO. This table contains sgRNAs that reached our threshold levels to be considered hits (log2FC > 1, FDR < 0.01). The sgRNAs targeting splicing sites, control sgRNAs (against non-targeting and non-essential genes), and other sgRNAs previously identified as essential were excluded from this list and they are also absent from the Figure 4b list.

| ***Gene-sgRNA ID*** | ***Targeted genomic region*** | ***log2FC*** | ***P.Value*** | ***FDR*** | ***Amino Acid Residues Affected*** | ***Predicted variants*** |
| --- | --- | --- | --- | --- | --- | --- |
| TERF2IP-69 | CCACAAGGCGTTACCGGTGA | 2.78875839 | 0.0000245933 | 0.002276314 | L163, W164 | L163S/P, W164R |
| TERF2IP-6 | CGTGGAGATGAAATCACCCG | 2.52592335 | 0.0002218155 | 0.007552321 | F78, I79, S80 | F78S, I79T, S80P |
| DKC1-9 | AACATACTCCTGCTTCCAGG | 1.79900892 | 0.0001296643 | 0.005920098 | Y416 | Y416H |
| TERF2IP-196 | CGTGGAGCGCAACGAGAGGC | 1.60222633 | 0.0000079884 | 0.001452705 | E89, N91 | E89G, N91D/S/G |
| ACD-65 | AAGCAGCGTGGCCCCGACGT | 1.5887237 | 0.0002809991 | 0.008677392 | L144 | L144P |
| BLM-296 | ATACAGCTTTTGGCCTACTT | 1.50750028 | 0.0000702430 | 0.004365735 | I1039, Q1040 | I1039M, Q1040R |
| TERT-61 | TGAACTTCTTGGTGTTCCTG | 1.41408157 | 0.0000103992 | 0.001501774 | F494 | F494S/L/P |
| TERT-273 | CAGGGGAATAGGCCGTGGGC | 1.38784591 | 0.0000030001 | 0.000920643 | F928, L927 | F928P, L927P |
| RTEL1-168 | ACGGCAAGCCTCTCCTCCTG | 1.32648119 | 0.0000073416 | 0.001441891 | L1177 | L1177P |
| TERT-93 | TGTGAACATGGACTACGTCG | 1.28292784 | 0.0000534360 | 0.003545549 | M636, N635 | M636V, N635S/D/G |
| TRF1-89 | CCGAGGCCGTGGCTGCCGGC | 1.186028 | 0.0000020444 | 0.000669193 | E71 | E71G |
| CTC1-330 | CAGAAGGGCCTTAGGGTCAA | 1.1674612 | 0.0002310439 | 0.00771718 | L572 | L572P |
| RTEL1-384 | CCACCTTCTTACGGCACAAG | 1.04584454 | 0.0000195901 | 0.002091032 | V191 | V191A |
| WRN-195 | ACAAGTACAAAAAGCCTCCT | 1.02568095 | 0.0001379939 | 0.006209401 | Q921, Q923, K924 | Q921R, Q923R, K924E |
| TERF2IP-80 | AAAAGTTTCTCAACCAGAGG | -1.0051184 | 0.0000250348 | 0.002276314 | K305 | K305R |
| RTEL1-231 | CCATCAGTGCTTACTATGCA | -1.0297245 | 0.0000097515 | 0.001501774 | I626, S627 | I626V, S627G |
| TINF2-11 | ATACACAGCAGACCTAGCCA | -1.0718988 | 0.0002206655 | 0.007552321 | T313, Y312 | T313A, Y312C |
| RTEL1-153 | AGCTTCGGAACACCTCCTAC | -1.082583 | 0.0000011103 | 0.000555391 | N128 | N128D/S/G |
| BLM-145 | ATCTATTTGCTCGCTTTCAG | -1.3392777 | 0.0000051047 | 0.00131917 | I218 | I218T |
| TRF1-83 | ATAAGCAATTTGCTTTTGAA | -1.362073 | 0.0001184852 | 0.005540594 | L214, L215 | L214S/P, L215P |
| TINF2-3 | AGCAATCCAAGCAATTCCTG | -1.4181733 | 0.0001497115 | 0.006229523 | C359, L357 | C359R, L357S/P |
| TRF2-241 | AAATGGGTAATTTTTAGAAA | -1.782338 | 0.0003154519 | 0.009219457 | F521, Y519 | F521L, Y519H |
